# Increased Axonal Bouton Stability during Learning in the Mouse Model of MECP2 Duplication Syndrome

**DOI:** 10.1523/ENEURO.0056-17.2018

**Published:** 2018-08-10

**Authors:** Ryan T. Ash, Paul G. Fahey, Jiyoung Park, Huda Y. Zoghbi, Stelios M. Smirnakis

**Affiliations:** 1 Department of Neurology, Brigham and Women’s Hospital and Jamaica Plain Veterans Administration Hospital, Harvard Medical School, Boston, MA 02115; 2 Medical Scientist Training Program, Baylor College of Medicine, Houston, TX 77030; 3 Department of Neuroscience, Baylor College of Medicine, Houston, TX 77030; 4 Department of Pediatrics, Texas Children’s Hospital and Baylor College of Medicine, Houston, TX 77030; 5 Department of Molecular and Human Genetics, Baylor College of Medicine, Houston, TX 77030; 6 Jan and Dan Duncan Neurological Research Institute at Texas Children’s Hospital, Houston, TX 77030; 7 Howard Hughes Medical Institute, Baylor College of Medicine, Houston, TX 77030

**Keywords:** autism, bouton, MECP2, motor learning, plasticity, synaptic

## Abstract

*MECP2* duplication syndrome is an X-linked form of syndromic autism caused by genomic duplication of the region encoding methyl-CpG-binding protein 2 (MECP2). Mice overexpressing *MECP2* demonstrate social impairment, behavioral inflexibility, and altered patterns of learning and memory. Previous work showed abnormally increased stability of dendritic spines formed during motor training in the apical tuft of primary motor cortex (area M1) corticospinal neurons in the MECP2 duplication mouse model. In the current study, we measure the structural plasticity of axonal boutons in layer 5 pyramidal neuron projections to layer 1 of area M1 during motor training. In wild-type littermate control mice, we find that during rotarod training the bouton formation rate changes minimally, if at all, while the bouton elimination rate more than doubles. Notably, the observed upregulation in bouton elimination with training is absent in *MECP2* duplication mice. This result provides further evidence of an imbalance between structural stability and plasticity in this form of syndromic autism. Furthermore, the observation that axonal bouton elimination more than doubles with motor training in wild-type animals contrasts with the increase of dendritic spine consolidation observed in corticospinal neurons at the same layer. This dissociation suggests that different area M1 microcircuits may manifest different patterns of structural synaptic plasticity during motor training.

## Significance Statement

Abnormal balance between synaptic stability and plasticity is a feature of several autism spectrum disorders, often corroborated by *in vivo* studies of dendritic spine turnover. Here we provide the first evidence that abnormally increased stability of axonal boutons, the presynaptic component of excitatory synapses, occurs during motor training in the *MECP2* duplication syndrome mouse model of autism. In contrast, in normal controls, axonal bouton elimination in L5 pyramidal neuron projections to layer 1 of area M1 more than doubles with motor training. The fact that axonal projection boutons get eliminated, while corticospinal dendritic spines get consolidated with motor training in layer 1 of area M1, suggests that structural plasticity manifestations differ across different M1 microcircuits.

## Introduction

The rewiring of synaptic connections in neural microcircuits provides a compelling mechanism for learning and memory throughout development and adult life (Chklovskii et al., 2004). Two-photon imaging of fluorescently labeled neurons has recently enabled the direct measurement of synaptic rewiring *in vivo*, revealing that new synapses form in motor cortex (M1) during motor training, and that the stability of these synapses correlates with how well the animal learns to perform the motor task (Xu et al., 2009; Yang et al., 2009). The layer 1 (L1) apical tuft dendritic spines that turn over during training receive inputs from a range of sources, including L2/3, L5, and L6 cortical pyramidal neurons, thalamocortical neurons, and others. It is currently not known how synaptic inputs from axonal projections to area M1 behave during training.

Experimental long-term potentiation and long-term depression (LTD) paradigms *in vitro* can induce axonal bouton formation and elimination (Antonova et al., 2001; Becker et al., 2008; Bourne et al., 2013). *In vivo*, axonal boutons are spontaneously formed and eliminated in adult sensory cortex (De Paola et al., 2006; Majewska et al., 2006; Stettler et al., 2006; Grillo et al., 2013), while behavioral training has been shown to alter bouton turnover in parallel fiber inputs to the cerebellum (Carrillo et al., 2013) and in orbitofrontal inputs to the medial prefrontal cortex (Johnson et al., 2016). In this work, we examine the turnover of boutons, the presynaptic component of synapses, in L5 pyramidal neuron axons that project to layer 1 of area M1.

Furthermore, we begin to assess whether training-associated plasticity in inputs to area M1 is altered in the *MECP2* duplication model of autism. *MECP2* duplication syndrome is caused by a genomic duplication that spans the methyl-CpG-binding protein 2 (*MECP2*) gene and leads to a progressive X-linked disorder of intellectual disability, autism, spasticity, and epilepsy (Ramocki et al., 2010). Overexpression of the *MECP2* gene in mice produces a similar progressive neurologic phenotype including autistic features (abnormal social behavior, anxiety, and stereotypies), spasticity, and epilepsy (Collins et al., 2004), and abnormal dendritic structure and plasticity (Jiang et al., 2013). Previous work found an increase in the formation and stabilization of dendritic spine clusters in apical dendritic tufts of corticospinal neurons in M1 (Ash et al., 2017) in these mice, pointing to a possible abnormal imbalance between synaptic stability and plasticity.

MeCP2 and other autism-associated proteins contribute to the development of mature axons and presynaptic structures (Antar et al., 2006; Belichenko et al., 2009; Degano et al., 2009; Chen et al., 2014; Garcia-Junco-Clemente and Golshani, 2014). Presynaptic electrophysiological function has been shown to be altered in *MECP2* duplication mice (increased paired-pulse facilitation; Collins et al., 2004) and other autism mouse models (Deng et al., 2013), and mice with mutations in the proteins mediating presynaptic plasticity often demonstrate autistic features (Blundell et al., 2010). LTD, a form of synaptic weakening that has a major presynaptic component (Collingridge et al., 2010), has been shown to be defective in several models of autism (D’Antoni et al., 2014). These findings implicate presynaptic dysfunction in autism, but, to our knowledge, axonal bouton structural plasticity has not been explored directly in a model of autism.

We measured axonal bouton structural plasticity in L1 of mouse M1 during rotarod training in the Tg1 mouse model of the *MECP2* duplication syndrome and compared with wild-type (WT) littermates. We found that the rate of bouton formation does not change significantly with rotarod training in either genotype, remaining approximately the same as the spontaneous bouton formation rate at rest. In contrast, the bouton elimination rate is dramatically accelerated during rotarod training in WT mice, whereas this effect is completely abolished in *MECP2* duplication mice. This supports the argument that increased synaptic stability manifests in the MECP2 duplication syndrome during training (Ash et al., 2017).

## Materials and Methods

### Animals

FVB-background *MECP2* duplication (*Tg1*) mice (Collins et al., 2004) were crossed to C57 thy1-GFP-M (Feng et al., 2000) homozygotes obtained from The Jackson Laboratory to generate male F1C57;FVB *MECP2* duplication;thy1-GFP-M mice and thy1-GFP-M littermate controls. All animal procedures were performed in accordance with the regulations of the Baylor College of Medicine animal care committee.

### *In viv*o 2-photon imaging

**Figure 1.**
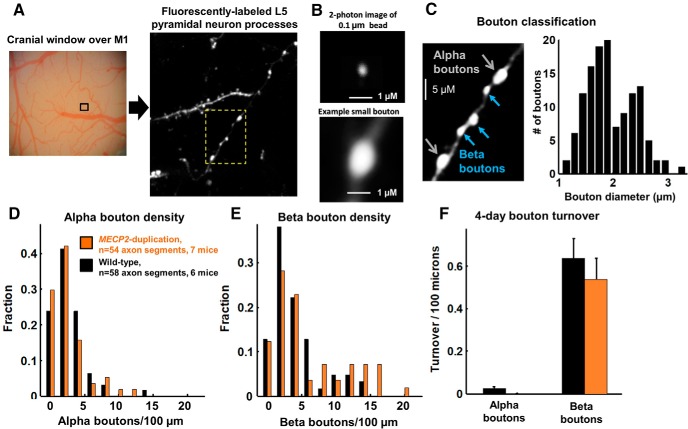
Bouton classification and density of L5 pyramidal neuron axonal projections to layer 1 of mouse primary motor cortex (M1). ***A***, *In vivo* 2-photon imaging. (1) A cranial window is drilled centered 1.6 mm lateral to the bregma to expose area M1. Correct localization to the forelimb was confirmed *post hoc* by electrical microstimulation (for review, see Ash et al., 2017). (2) GFP-labeled pyramidal neuron processes in layer 1 of area M1 are imaged. The yellow box is shown at high magnification in ***B***. ***B***, Top, The 2-photon image of a 0.1-μm-diameter fluorescent bead, revealing the resolving power of the microscope to be 0.4 µm full-width at half-maximum. Bottom, Example small (1.2-µm-diameter) bouton at the same magnification for comparison, showing that the resolution of the microscope allows ready discrimination of the boutons in this study. ***C***, Bouton classification. Left, Varicosities along axons are classified as alpha boutons (greater than ∼2 µm diameter, blue arrows) or beta boutons (1–2 µm diameter, yellow arrows) based on size (see Materials and Methods). Extraneous fluorescence structures masked for illustration purposes only. Right, Histogram of bouton diameters measured in a subset of axons (*n* = 54 alpha, *n* = 74 beta boutons), demonstrating a bimodal distribution. ***D***, ***E***, Histogram of densities of alpha (***D***) and beta (***E***) boutons per axonal segment in *MECP2* duplication mice (orange, *n* = 54 segments from seven mice) and WT littermates (black, *n* = 58 segments from six mice). ***F***, The 4 d spontaneous bouton turnover rate (boutons formed + boutons eliminated)/2*axon length, for alpha boutons and beta boutons. Alpha boutons were highly stable in this time frame.

All surgeries and imaging were performed blind to genotype. At least two weeks before the first imaging session (∼12-14 week-old-mice), a 3 mm-wide opening was drilled over motor cortex, centered at 1.6 mm lateral to bregma (Tennant et al., 2011), and a glass coverslip was placed over the exposed brain surface to allow chronic imaging of neuronal morphology (Mostany and Portera-Cailliau, 2008; Holtmaat et al., 2009; Mostany et al., 2013). Neural structures were imaged using a Zeiss *in vivo* 2-photon microscope with a Zeiss 20× 1.0 numerical aperture water-immersion objective lens. High-quality craniotomies had a characteristic bright-field appearance with well-defined vasculature and pale gray matter (Fig. 1*A*). Under 2-photon scanning fluorescent structures were reliably clear and visible with low laser power (<20 mW). A 0.1-μm-diameter fluorescent bead acquired with our 2-photon imaging setup is ∼0.4 μm full-width at half-maximum (Fig. 1*B*), confirming that our resolving power is sufficient to distinguish the 1- to 3-μm-diameter boutons we followed in the study. Only high-quality preparations (low background noise across all time points, <5 pixels, i.e., <0.5 µm slow motion artifact, <2 pixels; i.e., <0.2 µm fast motion artifact, and axons well isolated from other fluorescent structures) were used in the blinded analysis. Pyramidal neuron axons were imaged at high resolution (310 × 310 to 420 × 420 µm FOV; 0.1 µm/pixel; 1 µm *Z*-step size) to adequately capture individual boutons. Laser power was maintained at <20 mW (average, ∼10 mW) during image stack acquisition.

### Motor training

The Ugo Basile mouse rotarod was used for motor training. At least 2 h after imaging sessions, in the late afternoon, mice were placed on the rotarod, and the rotarod gradually accelerated from 5 to 80 rpm over 3 min. Single-trial rotarod performance was quantified as the time right before falling (16 cm fall height) or holding on to the dowel rod for two complete rotations without regaining footing. A 7-10 min rest period occurred between each trial. Four trials were performed per day.

### Analysis of bouton plasticity

Analysis was performed blind to genotype. Axons were chosen from the imaging field based on characteristic appearance, including the absence of dendritic spines, minimal branching, and the presence of synaptic boutons, as well as decreased width compared with dendrites. In the thy1-GFP M mouse line (Feng et al., 2000) that we used, the vast majority of GFP-labeled axons in the cerebral cortex arise from L5 pyramidal neurons, although occasional L2/3 and L6 pyramidal neurons and thalamocortical neurons may also be labeled (De Paola et al., 2006). Pyramidal neuron axons were targeted based on their thin shafts, high density of small (<3 µm diameter) en passant boutons, low tortuosity, and rare branching (type A3 axons), allowing them to be clearly distinguished from (1) L6 pyramidal neuron axons, which have high branching and a high density of terminaux boutons; and from (2) thalamocortical neurons, which have thicker axons and high branching (De Paola et al., 2006). Given the very sparse labeling of L2/3 neurons in the thy1-GFP M mouse line, we are confident that the great majority of axonal segments we imaged represent L5 pyramidal neuron projections to area L1 from other regions (i.e., chiefly from the premotor, the somatosensory, and the contralateral motor cortex; Hooks et al., 2013).

Segments of axon that were clearly visualized in all three time points were selected for analysis (length range, 30–360 µm; mean length, 138 µm). The presence of en passant boutons or terminaux boutons was noted by a blinded investigator, who further classified synaptic boutons as alpha (more than ∼2 µm or 20 pixels in diameter) or beta (less than ∼2 µm or 20 pixels in diameter). The threshold used for bouton classification was based on the bimodal distribution of boutons, separable at ∼2 µm diameter, present in the analyzed dataset (Fig. 1*C*; Grillo et al., 2013). The presence of a bouton was determined by a clear increase in axon diameter, increased fluorescence compared with the background axon, and the characteristic varicose contour determined by the judgment of an experienced investigator. In general, varicosities counted as boutons were >3 pixels (∼0.3 μm) wider than the axonal shaft diameter (corresponding to ∼2 SDs of the noise blur of the axonal shaft; Fig.1*B*) and more than twice as bright as the axonal backbone, as in the study by Grillo et al. (2013).

**Figure 2.**
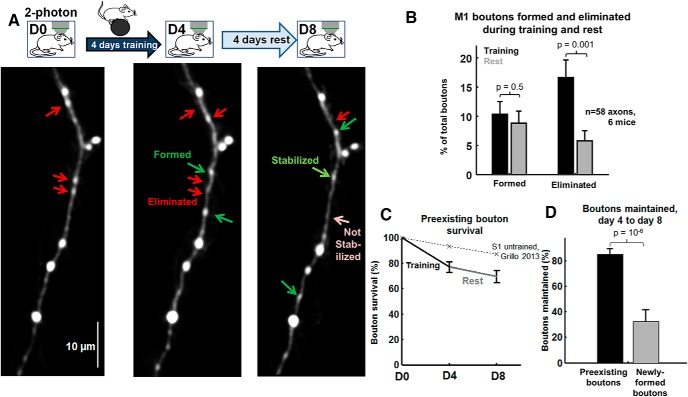
Bouton elimination increases during motor training in L1 of WT motor cortex. ***A***, Experimental paradigm and imaging time points. Sample images of axonal segments imaged before (left) and after (middle) 4 d of rotarod training to identify axonal bouton formation (green arrow) and elimination (red arrow) during training. Segments are imaged again following 4 d of rest (right) to identify boutons formed, eliminated, and maintained during rest and training-associated boutons that are stabilized (light green) or not stabilized (pink). Extraneous fluorescence structures were masked and image was slightly smoothed for illustration purposes only. ***B***, Bouton formation and elimination during training (black) and during rest (gray). Bouton elimination was significantly elevated during training, *p* = 0.001, *n* = 58 segments, Mann–Whitney *U* test. Total number of boutons studied: 314 baseline, 40 formed during training, 42 formed during rest, 64 eliminated during training, 23 eliminated during rest. Data were acquired from six mice. Statistics were performed across axonal segments. ***C***, Pre-existing bouton survival curves were across imaging days. The dotted line depicts baseline bouton survival and is calculated from the study by Grillo et al. (2013). ***D***, The fraction of boutons maintained during the rest period, measured for pre-existing boutons (present on day 0) that were still present on day 4 following training (black) and boutons formed during training (training-associated boutons, gray). *p* = 10^−6^, Mann–Whitney *U* test.

**Figure 3.**
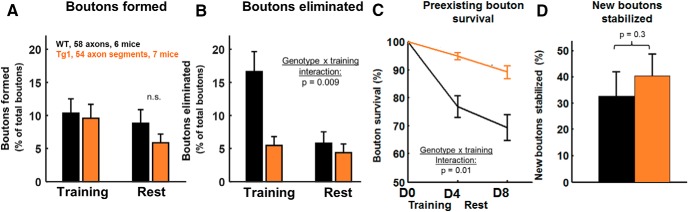
Increased stability of axonal boutons during training in *MECP2* duplication mice. ***A***, Bouton formation during training (training-associated boutons) and during rest in *MECP2* duplication mice and WT littermates. ***B***, Pre-existing bouton elimination during training and during rest in each genotype. ***C***, Pre-existing bouton survival curves across imaging. ***D***, The training-associated bouton stabilization rate, the number of boutons formed during training and still present after 4 d of post-training rest, is not significantly different across genotypes. Data are plotted as the percentage of boutons formed during training. Statistics: *A–C*, linear mixed-effects model ANOVA; *D*, Mann–Whitney *U* test.

Boutons located >50 µm away from the nearest other bouton were excluded from the analysis, so that stretches of bouton-free axon would not bias bouton density calculations. Four to 20 axons were analyzed from one to three imaging fields per mouse for 13 mice (6 WT, 7 *MECP2* duplication mice). Unless the investigator could clearly trace the continuity of axon segments, segments were analyzed as individual units. Although it is unlikely, the possibility cannot be completely excluded that, on occasion, more than one segment from a single axon was counted. Bouton formation and elimination (Figs. 2*B*, 3*A*,*B* ) was calculated as (boutons formed or boutons eliminated)/(total number of boutons observed across imaging sessions), analogous to the measure used in the study by Grillo et al. (2013). Bouton survival was calculated as the percentage of boutons identified at the first imaging time point that are present in subsequent imaging time points. Bouton stabilization was calculated as the percentage of newly formed boutons in the second imaging time point, which persisted in the third imaging time point.

### Statistics

Except where indicated, the Mann–Whitney *U* test was used for two-group statistical comparisons, and the linear mixed-effects models ANOVA was used for multigroup comparisons. The linear mixed-effects model ANOVA was instantiated with genotype and imaging time point as fixed effects, and mouse and axon implemented as random effects. This approximates a repeated-measures ANOVA for the two-way experimental design, accounting for any across-animal variability in determining statistical significance.

## Results

The Tg1 mouse model for *MECP2* duplication syndrome (FVB background) was crossed to the thy1-GFP-M mouse line (C57 background) to generate F1 hybrid males for experiments. A cranial window was placed over motor cortex (1.6 mm lateral to bregma) at 12–14 weeks of age, and at least 2 weeks following the surgery the mouse was placed under the 2-photon microscope to image GFP-labeled axons in layer 1 of area M1 (Fig. 1*A*; see Materials and Methods).

L5 pyramidal neuron axons are typically visualized as a thin string of fluorescence interspersed with fluorescent expansions or varicosities (en passant boutons) and rare spine-like terminaux boutons. They are readily differentiated morphologically from L6 neuron axons and thalamocortical axons (De Paola et al., 2006), which, in any case, are rarely fluorescent in these animals. The thy1-GFP M line primarily labels L5 pyramidal neurons in neocortex, and, therefore, the majority of axonal arbors we imaged are expected to arise from L5 of the somatosensory cortex, the premotor cortex, or the contralateral motor cortex, all of which project to L1 of area M1 (Colechio and Alloway, 2009; Mao et al., 2011; Hooks et al., 2013). Area M1 L5 neurons rarely send projections locally to layer 1 (Cho et al., 2004).

First, we report on axonal bouton structure and plasticity analyzed in littermate controls with normal *MECP2* expression. Axonal boutons were identified as periodic thickenings or extensions along the axon (Fig. 1*B*; see Materials and Methods). We observed a bimodal distribution of bouton sizes, the two modes separated at ∼2 µm diameter (Fig. 1*C*). These large (alpha) and small (beta) boutons were analyzed separately. The density of alpha boutons was 2.7 ± 0.3 boutons/100 µm (mean ± SEM; *n* = 58 axonal segments), and the density of beta boutons was 4.0 ± 0.4 boutons/100 µm (Fig. 1*D*,*E*), similar to the findings of a previous study (see Materials and Methods; Grillo et al., 2013). As expected given their large size (Grillo et al., 2013), alpha boutons were much more stable than beta boutons (Fig. 1*F*). Across 4 d of rest, the 4 d turnover rate [TOR = (gain rate + loss rate)/2] of alpha boutons was 0.5 ± 0.25% (0.02 ± 0.01 boutons/100 µm), while the TOR of beta boutons was 23 ± 4% (0.59 ± 0.08 boutons/100 µm). These results are comparable to those of a previous study in somatosensory cortex, which found a 0.1 ± 0.06% 4 d turnover for large boutons and a 30 ± 3% 4 d turnover for small boutons (Grillo et al., 2013, their Fig. 4*E*,*F*). Since alpha boutons were stable over time, hardly changing over the time course of the experiment, we restricted further analysis of structural plasticity to beta boutons.

**Figure 4.**
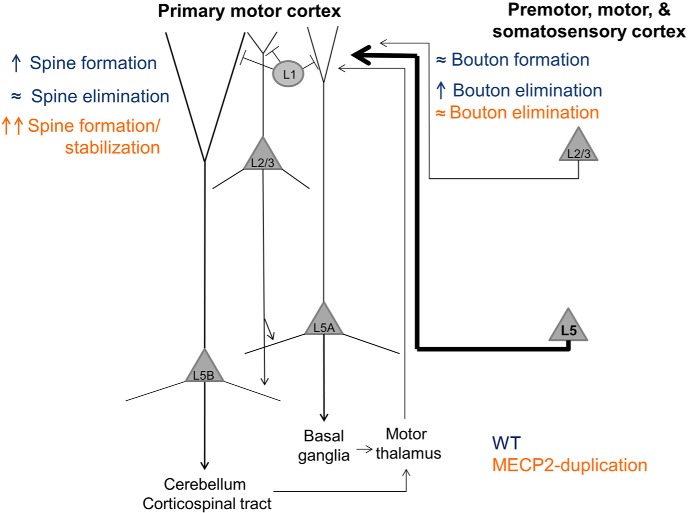
Sketch of structural plasticity phenotypes in dendrites and axonal projections in area M1 of *MECP2* duplication and WT mice. A highly simplified diagram of the layer 1 motor cortex circuit, including major local connections, inputs, and outputs. The imaged input projection is shown on the right in bold and represents axonal projections to L1 from L5 pyramidal neurons in somatosensory, premotor, and contralateral motor cortex. In WT mice (navy blue), spine formation increases in L5B neuron apical dendrites during motor training, while bouton elimination increases in L5 axonal projections. In *MECP2* duplication mice (orange), spine formation/stabilization increases even more than in WT mice during training, while bouton elimination is unchanged. See the text for details.

The experimental design is diagrammed in Figure 2*A*. L5 pyramidal neuron axonal projections to L1 of area M1 were initially imaged to identify baseline boutons. Then mice underwent 4 d of training on the accelerating rotarod task. Axons were reimaged to quantify training-associated bouton turnover. Mice rested in the home cage for 4 d, and axons were imaged again to observe bouton turnover during rest. WT mice performed progressively better on the rotarod across 4 d of training, as reported previously (Buitrago et al., 2004; Collins et al., 2004). Interestingly, rotarod training led to a dramatic increase in bouton elimination compared with rest: 17 ± 3% of total beta boutons were lost after 4 d of training compared with 6 ± 2% of total boutons lost after 4 d of rest (Fig. 2*B*; *p* = 0.001, Mann–Whitney *U* test; *n* = 58 axon segments from six mice). The bouton formation rate, in contrast, did not change significantly during motor training (Fig. 2*B*; training, 10 ± 2% of total boutons across time points; rest, 9 ± 2% of total boutons, *p* = 0.5). The measured formation rates and elimination rates were comparable to the spontaneous 4 d bouton formation and elimination rates previously observed in L5 pyramidal neuron axons in somatosensory cortex (formation, 8 ± 1%; elimination, 8.0 ± 0.2%; Grillo et al., 2013, their Fig. S4C,D). Overall, in control animals, motor training induces a doubling of bouton elimination in M1 without a concomitant change in the rate of bouton formation.

Plotting the survival fraction of pre-existing (“baseline”) boutons revealed that L5 pyramidal axons projecting to L1 of area M1 maintained 77 ± 4% of their baseline boutons (boutons present pretraining, on day 0) through 4 d of training (Fig. 2*C*). This value is significantly lower than prior estimates of spontaneous 4 d survival fractions of L5 pyramidal neuron axonal boutons (∼90% of baseline boutons; Fig. 2*C*, dotted line; De Paola et al., 2006, their Fig. 7B; Majewska et al., 2006, their Fig. 5; Grillo et al., 2013, their Fig. 3C).

Note that elimination rates (Fig. 2*B*) and survival curves (Fig. 2*C*) do not sum exactly to 100% because the elimination rate was calculated as a fraction of the total number of beta boutons observed across all time points to avoid outlier turnover rates in axons that had very few baseline boutons, following the study by Grillo et al., 2013 (see Materials and Methods).

We also compared the survival rate of newly formed training-related boutons with that of pre-existing boutons. In the 4 d of rest following training, 85 ± 4% of baseline pre-existing boutons (i.e., boutons present on day 0 that were also present on post-training day 4) were maintained, while newly formed boutons were maintained at a much lower rate of 32 ± 9% (Fig. 2*D*; *p* = 10^−6^, Mann–Whitney *U* test), which is consistent with the reported stabilization rate of spontaneously formed boutons in somatosensory cortex (newly formed, 35 ± 5% of all boutons over 4 d; Grillo et al., 2013).

We then assessed training-associated axonal bouton turnover in *MECP2* duplication mice. *MECP2* duplication mice performed significantly better on the rotarod than control littermates as described previously (Collins et al., 2004; Ash et al., 2017). The average length of analyzed axonal segments was not significantly different between mutants and WT littermates (WT mice, 142 ± 73 µm; *MECP2* duplication mice, 133 ± 73 µm; mean ± SD). The density of alpha boutons (Fig. 1*D*) and beta boutons (Fig. 1*E*) was also similar between the genotypes (alpha boutons: control, 2.7 ± 0.3 boutons/100 µm; *MECP2* duplication, 2.4 ± 0.3 boutons/100 µm; *p* = 0.4; beta boutons: control, 4 ± 0.4 boutons/100 µm; *MECP2* duplication, 5.8 ± 0.7 boutons/100 µm; *p* = 0.2, Mann–Whitney *U* test). Similar to WT mice, alpha boutons were highly stable compared with beta boutons in *MECP2* duplication mice (Fig. 1*F*).

Interestingly, the increased bouton elimination rate during training that we observed in WT mice did not occur in *MECP2* duplication mice (Fig. 3*B*). Significantly fewer boutons were eliminated during training in *MECP2* duplication mice (Fig. 3*B*; training, 5 ± 1% of total beta boutons; rest, 4 ± 1% of total boutons; *n* = 54 axon segments from seven mice) compared with littermate controls (training, 17 ± 3%; rest, 6 ± 2% of total boutons; *n* = 58 axon segments from six mice; effect of genotype: *t* = −2.9, *p* = 0.003; effect of training vs rest: *t* = −3.5, *p* = 0.0004; genotype × training interaction: *t* = 2.6, *p* = 0.009; linear mixed-effects models ANOVA, see Materials and Methods). Plotting the survival fraction of baseline (pretraining) boutons revealed that baseline boutons were significantly more stable in *MECP2* duplication mice versus littermate controls, especially during training (Fig. 3*C*; effect of genotype: *t* = −2.8, *p* = 0.004; effect of training versus rest: *t* = −3.1, *p* = 0.002; genotype x training interaction: t = 2.5, *p* = 0.01). *MECP2* duplication axons maintained 95 ± 1% of their boutons after 4 d of training, while control littermate axons maintained only 77 ± 4%. *MECP2* duplication axons lost a further 6 ± 1% of baseline boutons to reach 89 ± 2% bouton survival on day 8, while littermate controls lost a further 8 ± 2% to end at 69 ± 4%.

The rate of beta bouton formation was not significantly different between *MECP2* duplication mice and WT controls, neither during the training (Fig. 3*A*; control, 12 ± 2% of total boutons; *MECP2* duplication, 10 ± 2% of total boutons) nor during the rest phase (control, 9 ± 2% of total boutons; *MECP2* duplication, 6 ± 1% of total boutons; effect of genotype: *t* = 0.5, *p* = 0.6; effect of training vs rest: *t* = 0.4, *p* = 0.6; genotype × training interaction: *t* = −0.8, *p* = 0.4). The stabilization rate of newly formed boutons was also not significantly altered in *MECP2* duplication mice (40 ± 8%) compared with controls (32 ± 9%; Fig. 3*D*; *p* = 0.3). Again, note that elimination rates (Fig. 3*B*) and survival curve percentages (Fig. 3*C*) do not sum to 100%, as explained above, but note that the measured differences remain significant if the elimination rate is calculated as a fraction of baseline boutons instead of as a fraction of total boutons across time points (Fig. 3*C*).

Bouton formation, elimination, and stabilization rates did not correlate well with rotarod performance in individual animals for either genotype or pooled across genotypes (*p* > 0.05, *t* test on linear regression, all comparisons; data not shown), suggesting that other factors are potentially more important for the behavioral manifestations of motor learning.

## Discussion

The stability and plasticity of synaptic connections is a tightly regulated process that unfolds throughout life. A pathologic imbalance between stability and plasticity could lead to the altered patterns of learning and forgetting observed in autism mouse models (Collins et al., 2004; Rothwell et al., 2014) and in patients with autism (Treffert, 2014). In prior work (Ash et al., 2017), an abnormal increase in training-associated dendritic spine stability was found in the apical tuft of area M1 corticospinal neurons in the Tg1 mouse model of *MECP2* duplication syndrome. Here we investigated how axonal boutons in the L5 pyramidal neuron projection to L1 of primary motor cortex turn over during motor training in these animals. First, we find in WT mice that (1) the bouton formation rate is unaffected by motor training (Fig.2*B*) and (2**)** the bouton elimination rate more than doubles from ∼6% to ∼17% during training (Fig. 2*B*,*C*). In contrast, we find that the increase in training-associated bouton elimination observed in littermate controls does not occur in *MECP2* duplication mice (Fig. 3*B*), which exhibit increased bouton stability, particularly during training (Fig. 3*C*). The bouton formation rate during motor training was similar between *MECP2* duplication animals and littermate controls (Fig. 3*A*), and was not significantly different from the rate of bouton formation observed at rest in either genotype. A similar fraction of training-associated boutons was stabilized in both genotypes (Fig. 3*D*).

### Bouton formation and elimination with motor training in controls

Our spontaneous 4 d bouton turnover results are in agreement with those of a previous study of axonal bouton formation and elimination in L5 pyramidal neuron axons projecting to layer 1 of somatosensory cortex (Grillo et al., 2013), suggesting that baseline axonal bouton turnover in L1 is similar in sensory and motor areas. Here, we found that, in normal animals, the rate of axonal bouton elimination increases markedly during motor training in L5 pyramidal neuron projections to L1 of area M1, without a concomitant increase in the rate of bouton formation (Fig. 2*B*).

Grillo et al. (2013) performed *post hoc* electron microscopy reconstructions of nine axonal varicosities detected by 2-photon imaging and found that all nine boutons formed synapses, suggesting that the great majority of 2-photon imaging-identified boutons form a synapse. Our results therefore suggest that training leads to a weakening of L5 pyramidal inputs to layer 1 of area M1, at least as evidenced by structural analysis. Layer 5 axonal projections to L1 have several potential synaptic partners, including apical dendritic arbors of L5B corticospinal pyramidal neurons, L5A corticostriatal/corticocallosal neurons, L2/3 pyramidal neurons, and L1 interneuron dendrites (Fig. 4). Since L1 interneurons are sparse, most of the postsynaptic partners of the axonal boutons we studied are likely formed with one or more of the aforementioned classes of pyramidal neurons.

The increased elimination of presynaptic axonal boutons during training would then lead us to expect a corresponding loss in their postsynaptic partners (i.e., of dendritic spines located in the apical dendritic tufts of the target neurons). However, an increase in the formation rate of dendritic spines has been previously shown during motor training in the apical tuft terminal dendrites of L5 neurons in layer 1 of area M1 (Xu et al., 2009; Yang et al., 2009). This dissociation between L5 neuron dendritic spine formation and axonal bouton elimination during motor training suggests that the presynaptic partners of the L5 apical tuft dendritic spines studied previously during motor learning (Xu et al., 2009; Yang et al., 2009) arise from thalamocortical, L2/3, or L6 projections, which we did not study here. Indeed, projections to L1 of M1 from different brain areas and layers are known to preferentially target different cell types (Hooks et al., 2013).

Another nonexclusive possibility is that rather than connecting with a new axonal bouton, newly formed spines form a second synapse onto large pre-existing boutons already harboring a synapse. Evidence for this comes from correlative electron microscopy studies in the somatosensory cortex and hippocampus: ∼70% of newly formed spines synapse with a multisynapse bouton, compared to 20–30% of pre-existing spines (Knott et al., 2006; Nägerl et al., 2007; but, see also Woolley et al., 1996; Toni et al., 1999; Geinisman et al., 2001; Yankova et al., 2001; Federmeier et al., 2002; Nicholson and Geinisman, 2009; Lee et al., 2013). Dendritic spines formed during training may largely synapse on already existing, large, presynaptic boutons (alpha boutons in our study) where they compete with the previously present connections. Over time, some of these connections withdraw, re-establishing a new equilibrium that favors the new skill learning. Presumably, in the days to weeks following training, bouton formation modestly increases and/or bouton elimination decreases to bring bouton densities back to baseline levels. Overall, these results raise the interesting possibility that different pathways projecting to L1 of mouse area M1 may have different signatures of structural plasticity during motor learning.

### Increased bouton stability in *MECP2* duplication mice

We found that the training-associated increase in bouton elimination rate occurring in WT mice is abolished in *MECP2* duplication mice. The simplest interpretation of these results is that the L5 pyramidal neuron projection to L1 of area M1 undergoes less synaptic reorganization during training in mutants. In this case, the elevated synaptic turnover seen in mutant M1 (Ash et al., 2017) must be occurring in other L1 subcircuits (e.g., L2/3 or L6 pyramidal neuron projections). Increased bouton stability could also be due to more robust capture and stabilization of pre-existing boutons by newly formed training-associated spines, boutons that would have otherwise been eliminated due to loss of their prior postsynaptic targets during the training period (Knott et al., 2006; Nägerl et al., 2007). In this case, it would be possible to have accelerated reorganization in synaptic connectivity in the L5 pyramidal neuron to L1 circuit projection without any measurable change in the turnover of boutons. Imaging of bouton turnover in other projections to L1 of area M1 and quantification of multisynapse bouton density with and without training in mutants could address these two possibilities.

It is interesting to speculate that the training-associated bouton elimination that occurs in littermate controls is a natural end result of strong long-term depression (Becker et al., 2008; Wiegert and Oertner, 2013). In this case, the lack of bouton elimination in mutants may connote a disruption in processes regulating LTD. Taken along with the fact that abnormal LTD is observed in many other autism models (D’Antoni et al., 2014), it will be interesting to experimentally test whether LTD is indeed altered in M1 of *MECP2* duplication mice, and to see whether decreased LTD underlies the increased learning-associated bouton stability in the mutant.

### Relationship between bouton turnover and learning

The behavioral implications of increased L1 axonal bouton stability in mutants remain a matter of speculation. In our motor training experiments bouton elimination did not strongly correlate with behavioral performance either in mutants or controls, suggesting that other factors are potentially more important for the behavioral manifestations of motor learning. Prior work has shown that apical tuft L5 pyramidal neuron dendritic spine formation correlates with motor learning in normal animals (Yang et al., 2009), and *MECP2* duplication animals are known to exhibit increased spine formation and stabilization during learning (and at baseline) compared with wild-type littermates (Jiang et al., 2013; Ash et al., 2017).

We hypothesize that increased bouton survival during this period may in part reflect a higher rate of synapse stabilization, possibly due to an increased ability of *MECP2* duplication boutons to form synapses with newly generated spines. Although we have not proven it here, this increased ability may contribute to the faster and more durable learning that *MECP2* duplication animals exhibit in simple tasks like the rotarod and conditioned fear memory (Collins et al., 2004). Over time, however, the same process may restrict the overall flexibility of the motor circuit, leading to the motor deterioration phenotype observed at later ages.

### Potential limitations

It is important to note a number of limitations to the study. First of all, our quantification of presynaptic terminals depends entirely on morphologic measures. We used conservative criteria similar to those that in the hands of prior experimenters have been shown to reliably detect synapse-forming puncta (De Paola et al., 2006), and a 2-photon imaging study (Grillo et al., 2013) that systematically correlated bouton diameter with the presence of an EM-verified synapse found that all nine boutons they studied formed synapses, even the smallest, which had a diameter of ∼0.4 µm, considerably smaller than the boutons we studied here (diameter range, 1–3 µm; Fig. 1*C*). This suggests that the great majority of boutons we identify by 2-photon imaging form a synapse. Prior studies of bouton ultrastructure have estimated that ∼10% of varicosities do not form a synapse (Shepherd and Harris, 1998; White et al., 2004; Bourne et al., 2013), but, to our knowledge, these studies also included smaller varicosities and none related axonal varicosity size to the probability of a synapse.

Second, the rest phase occurred following training, so it is possible that some of the corresponding bouton turnover may reflect enduring consolidation processes that persist beyond training rather than a true rest phase. Having said that, the measured spontaneous axonal bouton formation and elimination are in very close agreement to those in previous studies (Grillo et al., 2013), suggesting that the measurements reflect baseline turnover.

Third, we cannot precisely determine the origin of the axonal afferents imaged in our study (Fig. 4). Some of the heterogeneity in plasticity observed across imaged axons could be due to projection-specific differences. For example, it would be interesting to speculate that the coarse sensorimotor training induced by the rotarod may drive greater bouton remodeling in somatosensory cortical inputs to area M1, while fine motor training requiring higher-order motor planning, such as the seed-grabbing task used by Xu et al. (2009), may induce greater remodeling in premotor cortical inputs.

Fourth, the postsynaptic partners of the imaged axons are unknown. The precise connectivity of inputs to M1, with S1 pyramidal neuron axons preferentially synapsing on L2/3 and L5A neurons and premotor cortex pyramidal neuron axons preferentially synapsing on L5B neurons (Mao et al., 2011; Hooks et al., 2013), enables a rich potential repertoire of synaptic reorganization during training. New methods targeting fluorescent proteins to specific input areas, as well as combinatorial techniques labeling presynaptic and postsynaptic partners (Kim et al., 2011; Druckmann et al., 2014), will be needed to tackle this question in the future.

### Conclusions and implications

In conclusion, we report here that L5 pyramidal neuron axonal projections to layer 1 of WT mouse motor cortex exhibit a selective escalation in bouton elimination during motor training, a plasticity process that is disrupted in the *MECP2* duplication syndrome mouse model of autism. These data constrain models of motor cortex plasticity underlying learning and underscore the possibility that different synaptic pathways within the cortical circuit may manifest different patterns of structural synaptic plasticity during learning. Future work studying plasticity along different synaptic pathways that link various areas along the motor circuit will shed further light on these issues. Finally, our results provide further evidence for an altered balance between the stability and plasticity of synaptic connections in favor of stability in the *MECP2* duplication syndrome mouse model.

## Synthesis

Reviewing Editor: Christian Hansel, University of Chicago Decisions are customarily a result of the Reviewing Editor and the peer reviewers coming together and discussing their recommendations until a consensus is reached. When revisions are invited, a fact-based synthesis statement explaining their decision and outlining what is needed to prepare a revision will be listed below. The following reviewer(s) agreed to reveal their identity: Peyman Golshani.

This manuscript is of potential interest, because it provides that the first description of an absence of learning-induced axonal pruning in a model of ASD/ID. Moreover, the study of a Mecp2 duplication will advance the field.

The authors use two-photon imaging to show that Rotorod learning-induced L5 neuron bouton elimination in L1 of motor cortex that is observed in wild-type mice is essentially absent in the MECP2 duplication mouse. These mice show enhanced Rotorod learning. The experiments are performed and data is analyzed carefully. Appropriate statistical techniques are used. While it is unclear whether this lack of bouton loss is causally related to the enhanced learning, the authors do not make that claim and simply present the finding. The discussion section does a good job of pointing out the limitations of the study. Nevertheless, the manuscript requires more work for consideration of publication in eNeuro.

Major comments:

1. The authors confirmed the observation that MECP2 duplication mice performed better than WT mice in rotarod training, then further found that MECP2 duplication mice exhibited higher bouton stability (minimal bouton elimination). However, the authors failed to provide any link between these two observations. In the discussion the authors admit that “the behavioral implications of increased L1 axonal bouton stabilization remain a matter of speculation”. The authors need to do better and at least provide a mechanistic framework of how increased bouton stability helps learning (if that's the reason why MECP2 duplication mice performed better). Otherwise, these two unrelated observations are presented out of context.

2. Further issues with the manuscript concern their methodology and data analysis.

The authors' main claim is that “the increased bouton elimination rate during training observed in WT mice did not occur in MECP2-duplication mice”. However, in both two-way ANOVA and mixed model for between-subject variance, there are significant genotype effects and training effects but only an almost significant (p=0.055) genotype x training effect, meaning that the bigger effect is that Mecp2 duplication mice generally eliminate less synapses and that training in general leads to more synapses elimination. These are the two claims the authors should have made instead.

Tukey correction is supposed to aid in comparisons between multiple levels of the same variable in the two-way ANOVA or selecting the significant interactions between variables with multiple levels. The authors seem to have used Tukey correction incorrectly to replace testing for the interaction effect, which was shown to not be insignificant result from the two ANOVA models. The authors could change their claim or alternatively choose a different analysis that lends more power to the interaction effect.

3. Another issue is that the authors used two-photon imaging to measure the shape and area of boutons and based their measurement on the resolution of acquired images.

The authors failed to discuss the resolving power of their optical system. The sizes of single boutons approach the resolving power of conventional microscopes as well as some two-photon systems, thus it is important the authors assure us that their system is powerful enough to be used to measure bouton shapes and sizes as claimed.

Minor comment:

Can the authors further expand their discussion to outline studies that looked at ultrastructure of the beta boutons specifically to know whether these smaller boutons were opposed to dendritic spines or postsynaptic densities? This would improve the paper. There must be older EM studies that have examined this in detail.

Overall, the authors showed some solid results regarding the bouton stability during motor learning in WT and MECP2 duplication mice, albeit with some methodological caveats and questionable significance, as mentioned above.
